# Alterations in Pregnenolone and Testosterone Levels in Male Shift Workers

**DOI:** 10.3390/ijerph20043195

**Published:** 2023-02-11

**Authors:** Massimo Bracci, Laura Zingaretti, Margherita Martelli, Raffaella Lazzarini, Gianmaria Salvio, Monica Amati, Marijana Milinkovic, Alfio Ulissi, Anna Rita Medori, Ermanno Vitale, Caterina Ledda, Lory Santarelli

**Affiliations:** 1 Occupational Health, Department of Clinical and Molecular Sciences, Polytechnic University of Marche, 60126 Ancona, Italy; 2 Occupational Medicine Unit, Management Staff Department, Marche University Hospital, 60126 Ancona, Italy; 3 Endocrinology Clinic, Department of Clinical and Molecular Sciences, Polytechnic University of Marche, 60126 Ancona, Italy; 4 Occupational Medicine Unit, Department of Medical and Surgical Specialties, Marche University Hospital, 60126 Ancona, Italy; 5 Section of Occupational Medicine, Department of Clinical and Experimental Medicine, University of Catania, 95124 Catania, Italy

**Keywords:** pregnenolone, testosterone, shift worker, night work, night shift work, shift work schedule, workplace, circadian rhythm, biological clocks, chronobiology disorders

## Abstract

Steroid hormone levels are closely related to the endogenous circadian rhythm induced by sleep–wake and dark–light cycles. Shift work that disrupts the circadian rhythm may influence the levels of steroid hormones. The association between shift work and alterations in female sex steroid hormone levels has been studied, but little is known about testosterone and its precursor pregnenolone levels in male shift workers. The present study investigated serum pregnenolone and testosterone levels in a group of shift and daytime male workers. All participants were sampled at the beginning of the morning shift. Lower levels of serum pregnenolone and total testosterone were found in the shift workers compared to the daytime workers. Variations in pregnenolone levels may have consequences for well-being, and they might produce consequences for the levels of hormones downstream of the steroid hormone cascade, such as testosterone. The low levels of testosterone found in shift workers demonstrate the perturbative effect of shift work on testosterone serum levels, which may be independent and/or related to pregnenolone synthesis.

## 1. Introduction

Shift work is a common work schedule, the prevalence of which has reached 21% among European countries, 29% in the US, and 36% in China, so there is an increasing interest in its potential effects on workers’ health [1,2,3]. Shift work can alter the endogenous circadian rhythm induced by sleep–wake and dark–light cycles [4]. Discrepancies between the endogenous rhythm and the exogenous rhythm can result in several health disorders, including alterations in the secretion of steroid hormones [4,5,6,7,8,9,10,11]. In fact, steroid hormones already have their own specific rhythm of secretion with a circadian and infradian periodicity [12]. Steroid hormones regulate many physiological processes and play a fundamental role in maintaining physical and mental health.

The association between shift work, including night shift work, and alterations in the levels of female hormones has been studied [13,14,15,16], and high levels of these hormones have been related to an increased risk of breast cancer [17,18]. In 2019, the International Agency for Research on Cancer (IARC) classified “night shift work” as “probably carcinogenic to humans” (Group 2A) [19,20]. Moreover, the National Toxicology Program (NTP), in the report of 2021, concluded that there is “high confidence for a causal relationship between human cancer and persistent night shift work—i.e., frequent and long-term night shift work, especially beginning in early adulthood—that causes circadian disruption” [21].

There are many factors that may play a part in excess breast cancer risk in shift workers [22,23,24,25,26], but alterations to the female sex hormone levels evidenced in female shift workers certainly play a role [19,21]. Similarly, male hormone levels may also be affected by shift work. Notably, the prostate is one of the organs most sensitive to fluctuations in the hormone testosterone [27]. In fact, the presence of testosterone is a fundamental requirement for prostate cancer cell growth, so the cornerstone of adjuvant hormone therapy in patients with advanced cancer is androgen deprivation [28]. Prostate cancer is the second most frequently diagnosed cancer in men worldwide. Despite the high frequency, little is known about the etiology of prostate cancer. Advancing age, a family history of prostate cancer, black race, and genetic susceptibility are proven risk factors for prostate cancer; body mass index (BMI), a fat-rich diet, hormone disorders, and physical inactivity are thought to be related too, but more studies are still needed to reach a definitive conclusion [29]. While night shift work is known to increase the risk of breast cancer, its association with prostate cancer is still controversial. The IARC in 2019 established that there is “suggestive evidence that risk of cancer of the prostate is positively associated with night shift work”, and the NTP in 2021 determined that there is “limited evidence of carcinogenicity of night shift work from studies in humans” concerning prostate cancer [19,21]. Since epidemiological studies are relatively few in number and are inconsistent, the association between shift work and prostate cancer cannot be confirmed with the available current data [30,31,32,33]. Several mechanisms have been proposed to explain the possible association between night shift work and prostate cancer risk, including circadian disruption and hormonal alterations [33,34,35,36].

Shift work can also affect male sexual function. In a large epidemiological study, Liu et al. reported significantly lower sperm counts in rotating shift workers compared with dayworkers [37]. These data have been recently confirmed by Demirkol et al., who investigated the effects of sleep disturbances on semen quality in men attending an infertility clinic, reporting a higher rate of oligozoospermia and poorer sleep quality in shift workers, with a significant correlation between sleep duration and sperm concentration [38]. Moreover, Rodriguez et al. reported worse erectile function in shift workers with shift work sleep disorders. Interestingly, they also reported that the negative effects of shift work on erectile function might be partially reversed by exogenous testosterone administration [39].

**Figure 1:**
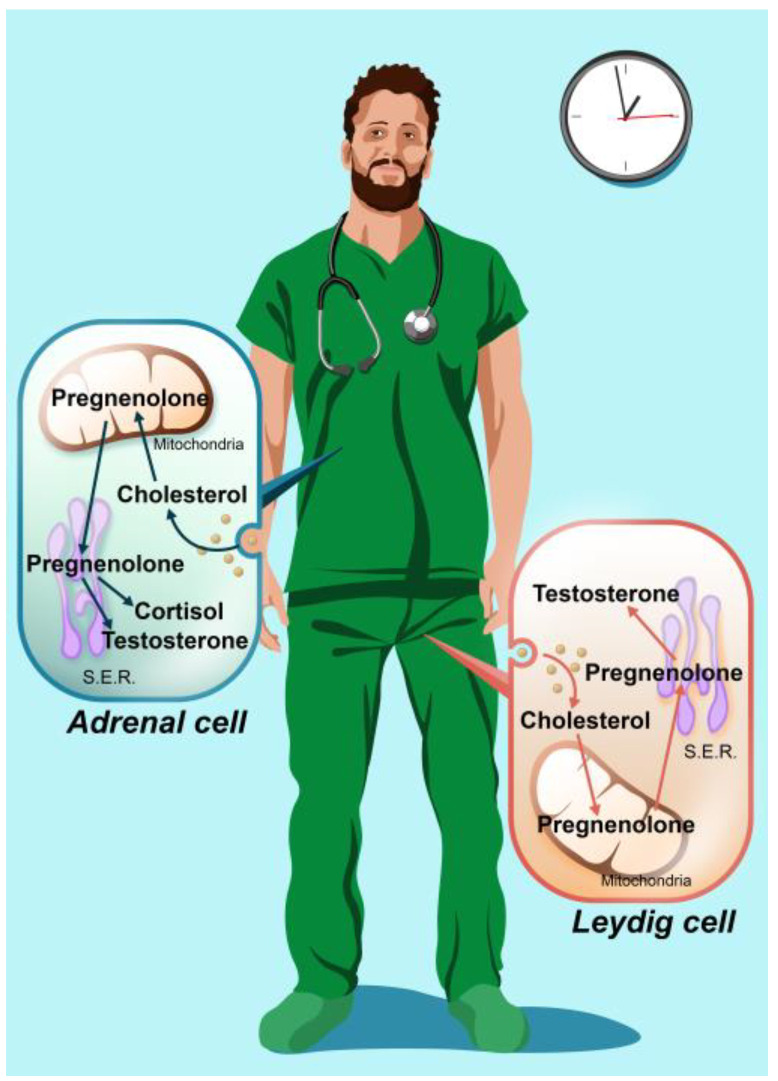
In males, steroid hormones are mainly produced in the adrenal and testicular Leydig cells. For both cell types, cholesterol is absorbed from the bloodstream and enters the mitochondria, where it is converted to pregnenolone and transferred to the smooth endoplasmic reticulum (S.E.R.). In the adrenal cell, pregnenolone is transformed into cortisol and, to a small extent, into testosterone. In the Leydig cell, pregnenolone is transformed into testosterone.

The synthesis of testicular steroid hormones involves the mitochondria, where pregnenolone is synthesized and then transformed into testosterone. Total testosterone travels in the blood mainly bound to proteins, and a small portion circulates as free testosterone. Cortisol levels are a marker of adrenal steroidogenesis since cortisol and testosterone initially share a common pathway involving pregnenolone but which leads to different hormone production [34] (Figure 1).

The aim of the study was to evaluate the influence of shift work on androgen levels in male shift workers, measuring their total and free testosterone and their precursor pregnenolone. In addition, their cortisol levels were investigated as a marker of adrenal steroidogenesis.

## 2. Materials and Methods

### 2.1. Participants and Sampling

A total of 100 participants (50 shift workers and 50 daytime workers) were enrolled among the physicians of the Regional Hospital of Ancona, Italy.

The shift workers had to be assigned for at least 2 years to shift work without any schedule breaks during the previous 2 months and must have worked at least 50 night shifts per year. The shift workers’ schedule was as follows: 8:00 A.M.–2:00 P.M., alternating with 2:00 P.M.–8:00 P.M. 6 days per week and 4–6 nights of 8:00 P.M.–8:00 A.M. per month.

Day workers were required to not have a history of shift work and to have had a routine sleep/wake schedule without sleepless nights for more than 3 weeks prior to the study. The working hours of the day workers were from 8:00 A.M.–2.00 P.M. 6 days a week.

Being performed as part of required, routine health surveillance, the study required no formal approval by the local ethics committee. However, the committee was consulted and gave informal permission. All workers were enrolled during the periodic medical check-ups required by Italian law. Workers provided their consent after receiving information about the purpose and procedures of the study, which was conducted according to the Helsinki Statement of Ethical Standards.

During the medical examination, the workers were evaluated and selected based on the following criteria: male, no personal or family history of prostate cancer, no ongoing drug treatment, negative history of endocrine disorders, psychiatric disorders, degenerative, or cardiovascular diseases, no insomnia, no chronic viral infections, no autoimmune diseases.

Pregnenolone and dehydroepiandrosterone (DHEA) are freely available as commercial compounds. The effects of exogenous administration of pregnenolone on male health are not clear but the oral ingestion of pregnenolone seems to lead to negligible modifications to the circulating sex steroids, such that it has not been included on the List of Prohibited Substances of the World Anti-Doping Agency (WADA) [40]. Conversely, DHEA administration is associated not only to a significant increase in circulating testosterone levels [41] but also with the up-regulation of androgen metabolites, including androstenedione, dihydrotestosterone, and estrone [42]. To avoid any possible interference, the assumption of commercial compounds containing pregnenolone or DHEA was investigated and excluded.

To avoid the acute alterations related to night shift, fasting venous blood samples were taken from both shift and daytime workers at the beginning of the morning shift after a regular night’s sleep on a day off. Blood samples were processed immediately after collection for the analysis of total cholesterol, triglycerides, and glycemia. Serum samples were divided into aliquots and were stored at −80 °C until hormone analysis.

### 2.2. Clinical Parameters Collection

Demographics, habit characteristics, and the measurement of anthropometric parameters were collected as part of the medical examination. Chronotype was assessed by the “Morningness–Eveningness Questionnaire” (MEQ) [43]—a questionnaire with 19 items and a total score ranging from 16 to 86 that is widely used in adults and workers [44,45,46]. Social jet lag has been investigated and computed as the absolute difference between midsleep on free days and midsleep on workdays [47]. All workers were sampled between 1 November and 15 December 2019 (local sunrise time is 6:40 A.M.–7:32 A.M., and local sunset time is 4:29 P.M.–5:00 P.M.) to limit the possible differences in time of sunlight exposure during the year. Evening exposure to blue light has been estimated considering the minutes of use of video display devices after dinner. Wake-up time on the day of blood sampling was investigated.

### 2.3. Hormones Assays

Serum samples stored at −80 °C were analyzed to determine cortisol, pregnenolone, and total and free testosterone levels within two months from sampling. Hormone levels were investigated using IBL ELISA enzyme immunoassays kits according to the manufacturer’s instructions (IBL America, Minneapolis, MN, USA). The ELISA assays have a standard range of 0–25 ng/mL for pregnenolone, 0–16 ng/mL for total testosterone, 0–60 pg/mL for free testosterone, and 0–800 ng/mL for cortisol. Optical density at 450 nm with 650 nm as the reference wavelength was determined using an ELISA microplate absorbance reader (Tecan Infinite F200, Tecan Group Ltd., Männedorf, Switzerland). All samples were measured in duplicate. Samples from each subject were assayed in the same batch. The inter- and intraassay variations of these analyses were all <10%.

### 2.4. Statistical Analysis

A total sample size of 90 participants was calculated a priori to detect significant differences with an effect size of 0.60, a power > 0.80, and an α = 0.05 (two-tailed) for all the variables studied. The theoretical sample size was increased by 10% in order to include a satisfactory final number of subjects. Data were expressed as mean ± SD. Student’s t-test was used to test differences in independent measures between the two groups. Dichotomous variables were expressed as percentages, and differences were analyzed by chi-square test. Pearson correlation test was used to analyze the relationships between the hormone levels. Multivariable linear regression analysis was used to assess the association between the type of work (shift work vs. daytime work: explanatory variable) and hormone level. BMI and “wake-up time on the blood sampling day” were considered as potential confounders a priori, and variables that were found to be statistically significant between the two groups were included in the multivariable analysis as additional confounders. Statistical significance was set at *p* < 0.05, and statistical tests were two-sided. Data analysis was performed with the Statistical Package for Social Sciences (version 19) software (SPSS Inc., Chicago, IL, USA).

## 3. Results

Of the 100 enrolled subjects, 12 were excluded from the study (n = 8 shift workers, n = 4 daytime workers) since they did not meet the selection criteria at the time of blood sampling. A total of 88 workers completed the study, of which 46 were daytime workers, and 42 were shift workers. There was a significant difference in age between the daytime and shift workers. The mean age of the day workers was 34.2 ± 5.4, while for the shift workers, it was 30.7 ± 5.5 (*p* = 0.003). Shift workers had a shift work seniority of 7.7 ± 5.1 and worked 5.6 ± 1.4 nights per month. There were no significant differences in smoking, BMI, physical activity, chronotype score, social jet lag, and minutes of use of video display devices after dinner between the two groups. Furthermore, no significant differences were found in serum cholesterol, triglycerides, and glucose levels between shift workers and daytime workers (Table 1).

**Figure 2:**
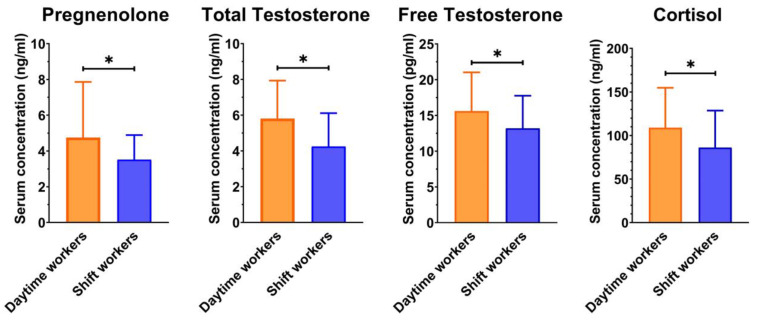
The mean serum concentrations ± SD of pregnenolone, total and free testosterone, and cortisol of daytime and shift workers. * = *p* < 0.05, student’s t-test for daytime vs. shift workers.

The daytime and shift workers were tested for differences in serum hormone levels; the shift workers showed decreased levels of pregnenolone as well as serum total and free testosterone when compared to daytime workers (3.5 ± 1.4 vs. 4.9 ± 3.1; 4.3 ± 1.9 vs. 5.8 ± 2.1, and 15.6 ± 5.4 vs. 13.2 ± 4.6, respectively). Cortisol originates from pregnenolone and provides one of the ways of transmitting the circadian message from the SCN to the peripheral tissues. Shift workers showed lower levels of cortisol compared to daytime workers (86.2 ± 42.4 vs. 109.0 ± 45.8, respectively; Figure 2).

The correlation between the serum concentrations of the tested steroid hormones in all subjects is good. All the concentrations of the tested hormones were correlated, except the total testosterone and cortisol levels (Table 2).

Since daytime and shift workers were significantly different in age, the association between the type of work (shift work vs. daytime work) and hormone levels was investigated by multivariable linear regression analysis, including age as a confounding independent variable. The multivariable linear regression analysis adjusted for age, BMI, and wake-up time on the blood sampling day, confirmed an association between the type of work and serum levels of pregnenolone and total testosterone (Table 3). Specifically, shift work was associated with low levels of pregnenolone (β −0.265; *p* = 0.036) and low levels of total testosterone (β −0.304; *p* = 0.008).

## 4. Discussion

Our results showed reduced levels of steroid hormones in the shift workers compared to the daytime workers. There were correlations between pregnenolone and total testosterone and between pregnenolone and cortisol, but not between total testosterone and cortisol, reflecting the common pathway during the first steps of steroidogenesis and divergence during the latter steps, which involve a different enzyme substrate specific to the adrenal and testis. Regarding the demographics and habit characteristics of the participants, our sample is homogeneous except for age. This finding was expected since the hospital management tends to allocate younger nurses to shift work [48,49,50]. Since age is a potential confounder, it was included as an independent variable in the multivariable analysis. The association between the type of work and serum levels of pregnenolone and total testosterone was significant in the multivariable analysis. Specifically, shift work was associated with low serum levels for pregnenolone and total testosterone. To the best of our knowledge, this is the first study that has investigated pregnenolone levels in shift workers.

Since the synthesis of pregnenolone is the first step of steroidogenesis taking place within mitochondria [51], our finding supports the thesis of crosstalk between mitochondrial function and circadian cycles [52,53]. Alterations in the synthesis of pregnenolone might produce consequences in the levels of hormones downstream of the steroid hormone cascade (e.g., testosterone and estrogens). Moreover, recent studies highlighted the direct involvement of pregnenolone on sperm function [54], suggesting that the impairment of pregnenolone production may have direct consequences on the reproductive health of male shift workers. Further studies on pregnenolone as a possible target of shift work and circadian disruption are encouraged.

Alterations to the total testosterone levels found in our sample of shift workers could be secondary to the low pregnenolone levels. In any case, our findings suggest a significant impairment of testicular steroidogenesis. This is in line with data reported in the literature, showing that sleep disturbances and circadian rhythm alterations negatively affect testicular function in terms of sperm parameters [55], erectile function [56], and testosterone production [57]. The finding of low levels of total testosterone in shift workers cannot exclude a possible association between shift work and prostate cancer. Epidemiological studies that have tried to link higher levels of testosterone to an increased risk of prostate cancer, providing conflicting results [27,28,58]. Testosterone levels show an inverse correlation with prostate volume in aging males [59], and even lower than normal levels of testosterone are sufficient to promote tumor growth [60]. The frequency of prostate cancer increases with age, which is usually accompanied by a progressive reduction in testosterone levels [61]. Other authors investigated the testosterone levels in shift workers or in simulated shift work. Toitou et al. studied testosterone levels in 12 shift workers, showing that testosterone peak and trough times were erratic and the serum concentrations were significantly lower in shift workers without any apparent phase shift [62]. Harding et al. found differences in acrophase during night shift compared to morning shift in a sample of 44 rotating shift workers [63]. After simulating the shift work in 14 healthy adults, the amplitude, mesor, and acrophase did not differ significantly between the day-shift and night-shift conditions [64]. Jensen et al. found that the diurnal rhythm completely followed the sleep–wake cycle and was not affected by the number of consecutive night shifts [65]. Papantoniou et al. found higher levels of mean 24 h urinary testosterone levels in a sample of 39 nights workers, but the differences were of borderline significance [35]. No difference in testosterone levels was found between shift and non-shift workers in a study demonstrating poorer erectile function in men who work night shifts [39]. No changes in salivary testosterone levels were found in 13 nightshift workers compared to 14 dayshift workers [66]. Recently, it was found that mimed shiftwork in rats reduced diurnal fluctuations of serum testosterone, and this was related to alterations in the transcription of the clock genes and mitochondria function induced by circadian desynchrony in Leydig cells [67].

The association between the type of work, the serum levels of free testosterone, and cortisol levels lacks significance in multivariable analysis, but it cannot be excluded. It is possible that the effect of shift work on free testosterone and cortisol levels is small in size, and the statistical power of our study did not allow us to evidence it. The serum levels of free testosterone are related to the total testosterone levels but also to other factors (e.g., plasma protein levels) that can reduce or mask the effect of the type of work on free testosterone. Regarding cortisol, it could be hypothesized that shift work has a more pronounced suppressive effect on testicular steroidogenesis than on adrenal steroidogenesis. The influence of shift work on cortisol levels has long been studied with conflicting results [62,68,69,70,71]. However, low cortisol levels were obtained by our research group on female shift nurses sampled at the beginning of the morning shift [22,72], and the present state of literature demonstrates that shiftwork, especially night shift work, significantly alters cortisol levels [9]. Moreover, the specific shift schedule and relatively few nights worked per month likely influenced the cortisol levels detected in our sample of shift workers.

Pregnenolone and total testosterone levels in shift workers need further investigation involving multiple samplings per day. Indeed, the concentration of pregnenolone and testosterone in blood follow a marked circadian rhythm with the highest levels in the morning [73,74,75]. Only with multiple blood samples during the day would it be possible to know whether the low values of pregnenolone and total testosterone in shift workers are due to a reduced amplitude of circadian oscillation, a phase shift, or a lower mesor. This constitutes a limitation of this study but, at the same time, the choice to evaluate a single morning sample made the study feasible and allowed for the adherence of an adequate number of subjects. 

Since pregnenolone and testosterone regulate several physiological processes, the evidence of reduced levels of these hormones obtained in this study may have important implications for the health of shift workers. Aging is associated with a constant decline in both endogenous pregnenolone [76] and testosterone [77] levels. Serum testosterone and its precursors, including pregnenolone, are lower in overweight and obese men compared with men with a normal BMI [78]. The symptoms and signs of low androgen levels include sexual dysfunction, infertility, reduced body hair, fatigue, gynecomastia, and altered body composition (increased body fat and decreased muscle mass) [77,79,80]. Moreover, low testosterone levels independently predict the risk of prostate cancer [81]. The relationship between androgens and physical fitness in a healthy population has not been extensively investigated, but a weak association between serum testosterone levels and greater aerobic capacity in healthy young men was reported [82]. The health issues associated with reduced levels of testosterone should be investigated in the context of the health surveillance of shift workers, and, in selected cases, an assessment of hormone levels is recommended. Conversely, there are no data available on the potential human health effects of endogenous pregnenolone levels, which therefore deserve further investigation. In shift workers, especially those who worked a high number of nights per month, the investigation of serum pregnenolone and testosterone hormone levels can be considered as part of the periodic health surveillance.

## 5. Conclusions

Our study showed lower serum levels of pregnenolone and total testosterone in a sample of male shift workers compared with daytime workers. This is likely to result in a negative effect on male sexual health, as suggested by in vivo and in vitro studies. It is not possible to relate low testosterone values to the risk of prostate cancer in shift workers; however, our results demonstrate a perturbative effect of shift work on testosterone serum levels. We cannot exclude the fact that the difference in total testosterone levels between the shift and daytime workers may be a consequence or at least related to the lower pregnenolone levels found in shift workers. The possibility of alterations in serum pregnenolone and/or testosterone levels should be taken into consideration in the health surveillance of shift workers.

## Figures and Tables

**Table 1:** Demographics, habit characteristics, and metabolic parameters of daytime and shift workers.

Parameters	Daytime Workers (n = 46)		Shift Workers (n = 42)		*p*-Value
	Mean ± SD	%	Mean ± SD	%	
Age (years)	34.2 ± 5.4		30.7 ± 5.5		0.003
Shift work seniority (years)			7.7 ± 5.1		
Night shift works (nights per month)			5.6 ± 1.4		
Smokers (%)		23.9		40.5	0.096
BMI (Kg/m^2^)	24.6 ± 3.4		25.1 ± 2.8		0.456
Physical activity (%)		60.9		51.5	0.422
Chronotype (MEQ score) *	54.1 ± 7.7		51.8 ± 6.9		0.145
Wake-up time on the blood sampling day	6:44 ± 0:40		6:32 ± 0:47		0.199
Social jet lag (minutes)	52.8 ± 47.1		51.8 ± 49.6		0.923
Use of video display devices after dinner (minutes)	76.4 ± 49.0		69.1 ± 42.5		0.459
Cholesterol (mg/dL)	174.9 ± 27.3		176.4 ± 34.5		0.820
Triglycerides (mg/dL)	87.4 ± 38.1		89.2 ± 47.0		0.843
Glucose (mg/dL)	84.2 ± 10.6		83.4 ± 8.4		0.698

* a chronotype higher score is indicative of “morningness” preference.

**Table 2:** Pearson correlation coefficients between pregnenolone, total and free testosterone, and cortisol in all subjects.

Parameters	Pregnenolone	Total Testosterone	Free Testosterone	Cortisol
Pregnenolone	1	0.26 *	0.33 **	0.25 *
Total testosterone	0.26 *	1	0.29 **	0.08
Free testosterone	0.33 **	0.29 **	1	0.25 *
Cortisol	0.25 *	0.08	0.25 *	1

* = *p* < 0.05, ** = *p* < 0.01.

**Table 3:** Association between the type of work (shift work vs. daytime work) and the serum levels of pregnenolone, total and free testosterone, and cortisol. Results of multivariable linear regression analysis adjusted for age, BMI, and wake-up time on the blood sampling day.

	Pregnenolone			Total Testosterone			Free Testosterone			Cortisol		
	R *	β	*p*-Value	R *	β	*p*-Value	R *	β	*p*-Value	R *	β	*p*-Value
Type of work (shift work vs. daytime work)	**0.31**	**−0.265**	**0.036**	**0.44**	**−0.304**	**0.008**	0.38	−0.123	0.285	0.36	−0.132	0.257

Statistically significant data in bold. * R of the model.

## Data Availability

The data supporting this study’s findings are available from the corresponding author upon reasonable request.
